# Urban Epidemic of Dengue Virus Serotype 3 Infection, Senegal, 2009

**DOI:** 10.3201/eid2003.121885

**Published:** 2014-03

**Authors:** Ousmane Faye, Yamar Ba, Oumar Faye, Cheikh Talla, Diawo Diallo, Rubing Chen, Mireille Mondo, Rouguiétou Ba, Edgard Macondo, Tidiane Siby, Scott C. Weaver, Mawlouth Diallo, Amadou Alpha Sall

**Affiliations:** Institut Pasteur, Dakar, Senegal (Ousmane Faye, Y. Ba, Oumar Faye, C. Talla, D. Diallo, M. Mondo, R. Ba, M. Diallo, A.A. Sall);; University of Texas Medical Branch, Galveston, Texas, USA (R. Chen, S.C. Weaver);; Laboratoire de Biologie Médicale BIO24, Dakar (E. Macondo, T. Siby)

**Keywords:** dengue, outbreak, Senegal, epidemic, dengue fever, dengue virus, viruses, DENV-3 genotype 3

## Abstract

An urban epidemic of dengue in Senegal during 2009 affected 196 persons and included 5 cases of dengue hemorrhagic fever and 1 fatal case of dengue shock syndrome. Dengue virus serotype 3 was identified from all patients, and *Aedes aegypti* mosquitoes were identified as the primary vector of the virus.

Dengue is an arboviral disease transmitted by *Aedes* spp. mosquitoes and caused by 4 serotypes of dengue virus (DENV): DENV-1, DENV-2, DENV-3, and DENV-4. DENV belongs to the family *Flaviviridae*, genus *Flavivirus* (*1*). More than 2.5 billion persons worldwide are considered at risk for dengue (*2*); the number of dengue infections per year has been estimated at 390 million, although only 96 million are symptomatic (*3*). 

Most dengue infections occur in urban areas in tropical and subtropical regions, but imported cases have been reported in nontropical regions. During October 2009, imported DENV-3 infections were diagnosed in Turin, Italy (*4*), and Marseille, France (*5*), from patients returning from the Louga and Thies regions in Senegal (Figure 1). DENV-specific IgM and/or RNA were also detected in 5 persons living in Dakar, Senegal, who were suspected to have dengue. We report results from investigation of the 2009 dengue epidemic in Senegal.

**Figure 1:**
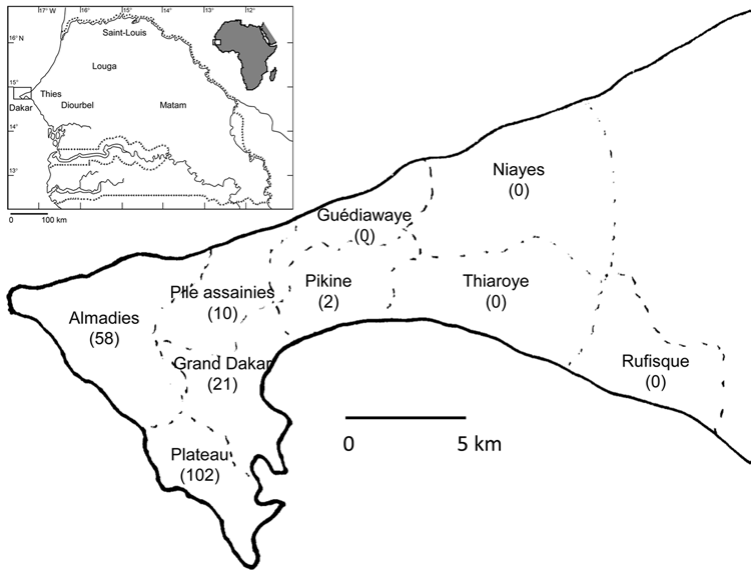
Geographic distribution of patients with confirmed dengue in the region of Dakar, Senegal. Number of patients is shown in parentheses. Inset shows location of Dakar in Senegal and in Africa.

## The Study

During October 2009–January 2010, a total of 696 serum samples were collected from persons in Senegal who were suspected to have dengue. A suspected dengue case was defined as fever and >2 of the following: myalgia, arthralgia, headache, and rash. Most samples were collected in Dakar (n = 606) and Thies (n = 87) (Table, Appendix). In Dakar, samples were collected from 9 neighborhoods: Plateau (n = 202), Almadies (n = 157), Grand Dakar (n = 117), Parcelles Assainies (n = 82), Pikine (n = 13), Guediawaye (n = 15), Niayes (n = 5), Thiaroye (n = 10), and Rufisque (n = 5) (Figure 1). Samples were tested by real-time reverse transcription PCR and ELISA for virus genome and IgM, respectively. 

**Table:** Epidemiologic and biological characteristics of suspected and confirmed dengue cases, Senegal, 2009*

Characteristic	Dakar									Thies	Louga	Other†	Total
	Pateau	Almadies	Grand Dakar	Parcelle	Pikine	Guediawaye	Niayes	Thiaroye	Rufisque				
Suspected cases	202	157	117	82	13	15	5	10	5	87	1‡	3	696
Patient sex													
M	91	60	56	44	8	10	2	6	2	38	1‡	3	320
F	111	97	61	38	5	5	3	4	3	49	0	0	376
Patient age, y, median (range)	30 (1–76)	29 (2–79)	38 (5–93)	28 (3–60)	25 (9–55)	29 (20–35)	33 (11–56)	40 (8–78)	45 (26–69)	30 (6–69)	32	53	
Health facility visited													
Private laboratory	144	125	64	40	2	0	5	0	5	0	1‡	0	385
Private clinic	23	10	0	0	1	0	0	0	0	0	0	0	34
Public health	35	22	53	42	10	15	0	10	0	87	0	3	277
Patient nationality													
Senegalese	96	98	89	82	13	15	5	10	5	87	1	3	504
Lebanese	86	11	1	0	0	0	0	0	0	0	0	0	98
French	13	33	6	0	0	0	0	0	0	0	0	0	52
Lusophone	5	14	20	0	0	0	0	0	0	0	0	0	39
Chinese	2	1	1	0	0	0	0	0	0	0	0	0	4
Confirmed cases	120	30	27	14	2	0	0	0	0	3	1‡	0	196
Patient sex													
M	56	12	12	8	0	0	0	0	0	1	1‡	0	89
F	64	18	15	6	2	0	0	0	0	2	0	0	107
Patient age, y, median (range)	32 (1–70)	26 (6–42)	44 (18–93)	26 (15–37)	18 (16–20)	NA	NA	NA	NA	42 (20–57)	NA	NA	
WHO disease classification													
DF	115	29	27	14	2	0	0	0	0	3	1‡	0	190
DHF	4	1	0	0	0	0	0	0	0	0	0	0	5
DSS	1	0	0	0	0	0	0	0	0	0	0	0	1
Laboratory testing conducted	47	10	9	NA	NA	NA	NA	NA	NA	NA	NA	NA	66
Leukopenia	34	7	5	NA	NA	NA	NA	NA	NA	NA	NA	NA	46
Thrombocytopenia	32	5	4	NA	NA	NA	NA	NA	NA	NA	NA	NA	41
Patient hospitalized	16	10	5	NA	NA	NA	NA	NA	NA	NA	NA	NA	31
Patient nationality													
Senegalese	46	14	12	14	2	0	0	0	0	2	1‡	0	90
Lebanese	63	2	0	0	0	0	0	0	0	0	0	0	65
Lusophone	3	5	12	0	0	0	0	0	0	0	0	0	20
French	7	9	3	0	0	0	0	0	0	1	0	0	20
Chinese	1	0	0	0	0	0	0	0	0	0	0	0	1

*Values are no. patients except as indicated. NA, not available; WHO, World Health Organization; DF, dengue fever; DHF, dengue hemorrhagic fever; DSS, dengue shock syndrome. †Diourbel, Saint Louis, and Matam. ‡Case identified and diagnosed in Italy.

In conjunction with human testing, mosquito sampling was performed during December 2009 in households with confirmed dengue cases, and entomologic risk indexes (i.e., Breteau and container indices [*6*]) were evaluated. DENV isolation and identification was performed on homogenized mosquitoes and human serum samples injected into mosquito cell lines, as described (*7*).

Partial DENV envelope protein coding regions were amplified by reverse transcription PCR (*8*), purified after electrophoresis from agarose gels, and sequenced for dengue serotype identification and phylogenetic analyses. A total of 196 (28.2%) suspected dengue cases were confirmed as dengue. Among confirmed case-patients, 45.9% (90/196), 33.0% (65/196), 10.0% (20/196), 10.2% (20/196), and 0.05% (1/196) belonged to the Senegalese, Lebanese, Lusophone, French, and Chinese communities, respectively; the Lusophone community includes persons from Cape Verde and Guinea-Bissau who live in Senegal. The ratio of confirmed to suspected cases was 17.8% (90/504), 66.3% (65/98), 51.2% (20/39), 38.4% (20/52), and 25.0% (1/4) for the Senegalese, Lebanese, Lusophone, French, and Chinese communities, respectively. 

Among the 196 confirmed case-patients, 31 were hospitalized; 5 were found to have dengue hemorrhagic fever (DHF), and 1 died of dengue shock syndrome. The M:F sex ratio for the case-patients was 0.83 (89 male, 107 female), and the median age was 31 years (range 1–93). 

The fatal case was in a 71-year-old Lebanese woman hospitalized in a private clinic in Dakar on October 30 with a 4-day history of fever; she had severe thrombocytopenia and elevated transaminase levels. Despite treatment (voluven and adrenaline), she died of cardiac arrest. Disseminated intravascular coagulation was probably responsible for the hemorrhagic syndrome. 

Of the 5 other patients (4 Lebanese and 1 Senegalese) who had hemorrhagic manifestations, all had fever, epistaxis, and melena (5/5) associated with thrombocytopenia (platelet count 50–90 × 10^9^/L) and leukopenia (leukocyte count 2.5–3.5 × 10^9^ cells/L); 4 reported headache, and 3 reported myalgia/arthralgia. All 5 patients were hospitalized and received transfusions of fresh frozen plasma, platelet concentrates, and other supportive treatments. Dengue-specific IgM and/or RNA were detected in serum samples collected 2 or 8 days after the onset of symptoms. All patients recovered and were discharged from the hospital after 8 or 10 days. 

**Figure 2:**
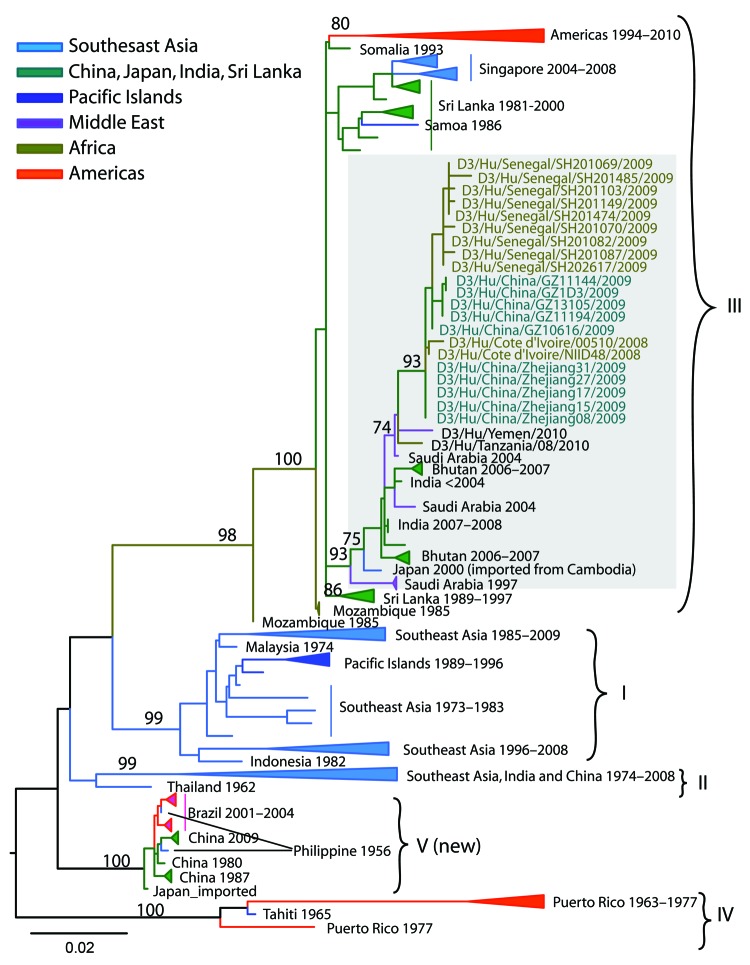
Maximum-likelihood phylogenetic tree of dengue virus serotype 3 (D3) sequences from Senegal compared with other sequences. The tree was constructed on the basis of an 885-bp segment of the envelope protein gene. Bootstrap values >70 are labeled next to the node. Sequences from different geographic areas are shown by different colors. Gray shading indicates sequences from Senegal and closely related strains. Scale bar indicates nucleotide substitutions per site.

Using mosquito continuous cell lines, we recovered 49 DENV-3 isolates from confirmed case-patients. Phylogenetic analysis of sequences from mosquito and human samples revealed that DENV-3 genotype III closely related to isolates circulating in Côte d’Ivoire (2008) and China (2009) was circulating during the Senegal outbreak (Figure 2). Dates of illness onset of confirmed cases indicated that the outbreak started in late September 2009, peaked in mid-November, and declined in mid-December. 

The entomologic investigation found high epidemic risk in all localities infested with DENV vectors. The Breteau index ranged from 6.6 to 195.2 in Dakar, 1.6 to 32.7 in Louga, and 1.1 to 14.9 in Thies, whereas the container indices ranged from 15 to 63.2 in Dakar, 5.3 to 15.2 in Thies, and 14.3 to 64.2 in Louga. A total of 5,730 mosquitoes were collected; these belonged to 8 species: *Aedes aegypti*, *Anopheles gambiae, Culex quinquefasciatus, Cx. tigripes, Cx. tritaeniorhynchus, Cx. antennatus, Cx. ethiopicus,* and *Cx. nebulosus*. Most mosquitoes were collected as adults, but 1,675 emerged from larvae that were collected in the field. *Cx quinquefasciatus* mosquitoes predominated, but *Ae. aegypti* mosquitoes, the only dengue vector collected, were found in all sites. DENV-3 was detected from 3 pools of mosquitoes collected in 2 neighborhoods of Dakar: Plateau (2 pools) and Parcelles Assainies (1 pool).

## Conclusions

An epidemic of DENV-3 occurred in Senegal during September–December 2009; of the 196 laboratory-confirmed cases, most (193) occurred in Dakar. This finding could indicate that transmission rates were higher in this urban area but may have been the result of bias in sample collection; dengue surveillance was less active at health facilities in in other regions, which provided only 277 (39%) of the 696 samples collected from persons who were suspected to have dengue. Fever, headache, myalgia, vomiting, thrombocytopenia, and leukopenia were the most frequent signs and symptoms among patients with confirmed dengue, as described (*9*).

The proportion of DHF cases in this outbreak seemed to be high at 3% when compared with previous reports in the Americas from the 1980s through 2007, in which DHF rates ranged from 1.3% to 2.4% (*10*). However, our sample size was limited and lacks confirmatory power. 

The percentage of confirmed dengue cases among suspected cases in different communities showed that persons in Senegalese communities were significantly less affected than those in Leebanese (χ^2^ = 98.3, df = 1; p<0.0001), Lusophone (χ^2^ = 23.3, df = 1; p<0.0001), and French (χ^2^ = 11.3, df = 1; p<0.001) communities. Moreover, 5 (83.3%) of the 6 cases with hemorrhagic manifestations occurred in the Lebanese community, which suggests that disease severity might be associated with community exposure. 

*Ae. aegypti* mosquitoes were the most likely vector of DENV-3 transmission during this epidemic, given their association with DENV and the overlap of their distribution and abundance with the locations of dengue confirmed cases. *Ae. aegypti* mosquitoes are known to be a competent vector for DENV in West Africa (*11*) and could maintain DENV-3 through vertical transmission in Senegal, as described (*12*). The absence of suspected or confirmed dengue cases in Louga, despite the high density of *Ae*. *aegypti* mosquitoes, suggests that the first reported case-patient contracted the DENV infection elsewhere, possibly in Dakar, which he had visited several times.

The phylogenetic analysis of DENV-3 strains isolated during the outbreak suggests that they belong to genotype III and are closely related to DENV-3 isolated from Côte d’Ivoire and China in 2008 and 2009, respectively (*13*). Hence, the strain responsible for this outbreak may have been introduced into Senegal by travelers from Asia or from Côte d’Ivoire. 

Our findings suggest that increased urban dengue activity is plausible in Senegal. Given ongoing population growth, explosive urbanization, infrastructure building, and international travel, dengue surveillance and preparedness should be reinforced. Furthermore, phylogenetic studies incorporating more DENV-3 strains would shed light on the origins of this DENV-3 outbreak. 

## References

[1] Lindenbach B, Thiel H, Rice C. Flaviviridae: the virus and their replication. In: Knipe DM, Howley PM, editors. Fields virology, 5th ed. Philadelphia: Lippincott, Williams & Wilkins; 2007. p. 1101–52.

[2] World Health Organization. Dengue: guidelines for diagnosis, treatment, prevention, and control. Geneva. Organization. 2009;•••:174. 23762963

[3] Bhatt S, Gething PW, Brady OJ, Messina JP, Farlow AW, Moyes CL, The global distribution and burden of dengue. Nature. 2013;496:504–7 . 10.1038/nature12060 23563266 PMC3651993

[4] Nisii C, Carletti F, Castilletti C, Bordi L, Meschi S, Selleri M, A case of dengue type 3 virus infection imported from Africa to Italy, October 2009. Euro Surveill. 2010;15:19487 . 20184855

[5] Franco L, Di Caro A, Carletti F, Vapalahti O, Renaudat C, Zeller H, Recent expansion of dengue virus serotype 3 in West Africa. Euro Surveill. 2010;15:19490 . 20184854

[6] Lok CK. Methods and indices used in the surveillance of dengue vectors. Mosquito Borne Diseases Bulletin. 1985;1:79–81.

[7] Digoutte JP, Calvo-Wilson MA, Mondo M, Traore-Lamizana M, Adam F. Continuous cell lines and immune ascitic fluid pools in arbovirus detection. Res Virol. 1992;143:417–22 . 10.1016/S0923-2516(06)80135-4 1297177

[8] Gaunt MW, Sall AA, De Lamballerie X, Falconar AI, Dzhivanian TI, Gould AG. Phylogenetic relationships of flaviviruses correlate with their epidemiology, disease association and biogeography. J Gen Virol. 2001;82:1867–76 . 11457992 10.1099/0022-1317-82-8-1867

[9] Aggarwal A, Chandra J, Aneja S, Patwari AK, Dutta AK. An epidemic of dengue hemorrhagic fever and dengue shock syndrome in children in Delhi. Indian Pediatr. 1998;35:727–32 . 10216566

[10] San Martín JL, Brathwaite O, Zambrano B, Solórzano JO, Bouckenooghe A, Dayan GH, The epidemiology of dengue in the Americas over the last three decades: a worrisome reality. Am J Trop Med Hyg. 2010;82:128–35 . 10.4269/ajtmh.2010.09-0346 20065008 PMC2803522

[11] Vazeille M, Yébakima A, Lourenço-de-Oliveira R, Andriamahefazafy B, Correira A, Rodrigues JM, Oral receptivity of *Aedes aegypti* from Cape Verde for yellow fever, dengue, and chikungunya viruses. Vector Borne Zoonotic Dis. 2013;13:37–40 . 10.1089/vbz.2012.0982 23199267

[12] Joshi V, Mourya DT, Sharma RC. Persistence of dengue-3 virus through transovarial transmission passage in successive generations of *Aedes aegypti* mosquitoes. Am J Trop Med Hyg. 2002;67:158–61 . 12389940 10.4269/ajtmh.2002.67.158

[13] Sun J, Lin J, Yan J, Fan W, Lu L, Lv H, Dengue virus serotype 3 subtype III, Zhejiang Province, China. Emerg Infect Dis. 2011;17:321–3 . 10.3201/eid1702.100396 21291623 PMC3204750

